# Safety, Feasibility, and Acceptability of the PrePex Device for Adult Male Circumcision in Malawi

**DOI:** 10.1097/QAI.0000000000000774

**Published:** 2016-05-24

**Authors:** Pamela K. Kohler, Beth A. Tippett Barr, Anderson Kang'ombe, Carola Hofstee, Franklin Kilembe, Sean Galagan, David Chilongozi, Dorothy Namate, Medson Machaya, Khuliena Kabwere, Mwawi Mwale, Wezi Msunguma, Jason Reed, Frank Chimbwandira

**Affiliations:** * Department of Global Health, University of Washington, Seattle, WA;; † Department of Psychosocial and Community Health, University of Washington, Seattle, WA;; ‡ Health Services, US Centers for Disease Control and Prevention, Lilongwe, Malawi;; § Banja La Mtsogolo, Blantyre, Malawi;; ‖ Médecin Sans Frontières, Nsanje, Malawi;; ¶ International Training and Education Center for Health, Lilongwe, Malawi;; # Malawi Ministry of Health, Lilongwe, Malawi;; ** US Centers for Disease Control and Prevention, Atlanta, Georgia; and; †† Jhpiego, An affiliate of Johns Hopkins University, Washington, DC.

**Keywords:** voluntary medical male circumcision, HIV prevention, nonsurgical devices, PrePex

## Abstract

**Introduction:**

Nonsurgical adult male circumcision devices present an alternative to surgery where health resources are limited. This study aimed to assess the safety, feasibility, and acceptability of the PrePex device for adult male circumcision in Malawi.

**Methods:**

A prospective single-arm cohort study was conducted at 3 sites (1 urban static, 1 rural static, 1 rural tent) in Malawi. Adverse event (AE) outcomes were stratified to include/exclude pain, and confidence intervals (CIs) were corrected for clinic-level clustering.

**Results:**

Among 935 men screened, 131 (14.0%) were not eligible, 13 (1.4%) withdrew before placement, and 791 (84.6%) received the device. Moderate and severe AEs totaled 7.1% including pain [95% CI: 3.4–14.7] and 4.0% excluding pain (95% CI: 2.6 to 6.4). Severe AEs included pain (n = 3), insufficient skin removal (n = 4), and early removal (n = 4). Among early removals, 1 had immediate surgical circumcision, 1 had surgery after 48 hours of observation, 1 declined surgery, and 1 did not return to our site although presented at a nearby clinic. More than half of men (51.9%) reported odor; however, few (2.2%) stated they would not recommend the device to others because of odor. Median levels of reported pain (scale, 1–10) were 2 (interquartile range, 2–4) during application and removal, and 0 (interquartile range, 0–2) at all other time points.

**Conclusions:**

Severe AEs were rare and similar to other programs. Immediate provision of surgical services after displacement or early removal proved a challenge. Cases of insufficient skin removal were linked to poor technique, suggesting provider training requires reinforcement and supervision.

## INTRODUCTION

Voluntary medical male circumcision (VMMC) has been shown to decrease risk for acquisition of HIV among heterosexual men by approximately 60%,^1–3^ prompting a large-scale effort to expand VMMC services in countries with low numbers of circumcised men and high HIV prevalence.^4^ Malawi, 1 of 14 target countries for circumcision scale-up, has an estimated HIV prevalence of 12.5%, with male circumcision prevalence estimated at 20.7%.^5^ The Government of Malawi formally adopted its male circumcision policy in 2011 and was expected to complete more than 2 million procedures by the end of 2015. However, by 2012, Malawi had reached <2% of this target.^6^

Countries with limited health infrastructure face a number of challenges in scaling up VMMC services, including inadequate financial resources, lack of surgical infrastructure, and lack of human resource capacity.^7^ Nonsurgical circumcision devices present an alternative to surgery, where health infrastructure and resources are limited.^8^ PrePex (Circ MedTech), one of such devices, works by compressing the foreskin with 2 rings to block circulation distally, after which the foreskin becomes necrotic and is removed after a period of 7 days. Advantages of the device are that the procedure does not require injectable anesthesia, suturing, or a sterile setting, and the device can be safely placed and removed by lower cadre providers.^9^

Early clinical studies indicated that the PrePex device had similar adverse event (AE) rates to surgical circumcision; however, a somewhat longer healing time was documented.^10–12^ After the clinical trials, the World Health Organization (WHO) prequalified the device for consideration in country HIV prevention programs and recommended a series of noncomparative field studies in settings of intended use.^13^ These field studies aim to aid ministries of health in deciding whether and how to incorporate the PrePex device into their national VMMC programs, by assessing implementation within their local context. This study was the pilot implementation study for Malawi and aimed to assess the safety, feasibility, and acceptability of the PrePex device for adult male circumcision in Malawi.

## METHODS

### Study Design

A prospective single-arm study was conducted at 3 sites (1 urban static, 1 rural static, 1 rural tent) in Malawi. Before study start, surgical VMMC providers were trained in PrePex placements, removal, and clinical care by master trainers from Rwanda.

Male clients were seen at 6 visits over a period of 42 days. Devices were placed on day 0, clients returned for a clinical examination on day 2 or day 4 depending on clinic hours, and for removal on day 7. Follow-up clinical examinations were conducted at days 14 and 42, and participants returned for an in-person survey on day 28. Clients were encouraged to return to clinic in the event of any concerns or problems.

### Population and Setting

Provider participants and male clients were recruited between April and September 2014 from 3 sites linked to ongoing clinical services in Malawi: an urban static site in Lilongwe District, a rural static site in Nsanje District, and a rural tent site in Mulanje District. The Lilongwe and Mulanje programs offered surgical VMMC services. In Nsanje, VMMC was only available through the general operating theatre at the district hospital. The Lilongwe outreach site operated out of a VMMC clinic located on hospital premises, the Mulanje tent site was located near a health centre and district hospital and remained in place for the study duration, and the Nsanje site operated out of renovated clinic space within the district hospital compound.

### Recruitment and Inclusion Criteria

Twelve midlevel providers, 4 from each site, were trained and enrolled. Provider participants included 1 registered nurse, 6 clinical officers, 4 nurse midwife technicians, and 1 medical assistant. All were practicing surgical circumcision providers through the ministries of health or nongovernment organizations.

In Lilongwe and Mulanje, male clients were recruited among men voluntarily presenting for surgical circumcision. At these sites, men were informed about both surgical and PrePex services and were offered a choice of circumcision approaches. In Nsanje, study team members conducted outreach with local stakeholders and village chiefs to raise awareness about male circumcision for HIV prevention and the study. Eligible men across all sites were HIV-negative, uncircumcised, age 18–49, who reported access to a mobile telephone most times of the day. Exclusion criteria included general medical conditions, genital anatomic abnormalities, active genital infections, inability to fit one of the available PrePex device sizes, and evidence of partial circumcision or scarification.

### Sample Size Calculations

A sample size of 805 males was powered to detect the occurrence of AEs, estimated from the Rwanda trials at 2%.^10,11^ Sample size estimation was based on a confidence interval (CI) of 95%, and a margin of error of ±1.5%. To account for between- site variation and within- site clustering, a design effect of 2.0 was used. In addition, inflation of 20% in the sample size was used to account for loss to follow-up.

### Data Collection

The study assessed provider outcomes of acceptability and client outcomes of safety and acceptability. Provider participants completed 2 surveys, 1 at study midpoint and 1 at study end. Client safety outcomes assessed the proportion of moderate, severe, and device-related AEs among all placements. Timing, type, relatedness to the procedure, and resolution of each AE were documented. President's Emergency Plan for AIDS Relief (PEPFAR) AE definitions were used, which determine severity based on the intervention required (mild require minimal monitoring or hygiene, moderate require antibiotic medication or suturing, severe require surgical completion). Relatedness was classified as not related, possibly related, or definitely related.^14^ Participants who did not return for scheduled visits were telephoned and asked to return to clinic. When clients still wearing the device were unable to be contacted after 3–5 calls, a study team member visited the client's place of residence. Participants with AEs requiring surgical intervention were offered medical follow-up; however, they were withdrawn from further surveys and procedures. Their AE information was included in study outcomes.

### Statistical Analysis

Statistical analyses were conducted in Stata version 11.2 (College Station). Proportions of AEs were calculated, and 95% CIs were generated using generalized linear models with a Poisson distribution, controlling for clustering at the facility level. Median and interquartile ranges (IQRs) of reported visual analogue scale pain scores were calculated for each time point and presented as box plots where 0 represents no pain and 10 represents the most pain. AE outcomes were stratified to include and exclude pain to allow comparison with previous studies, where pain was not considered an AE. Device-related events, including displacement and self-removal or early removal, whereas considered severe events as a result of the need for surgery, are presented separately.

### Ethical Considerations

Ethical approvals were received by the Malawi National Health Sciences Research Committee (#1129), the Médecins Sans Frontières Ethics Review Board (#1331), the University of Washington Institutional Review Board (#45648), and the US Centers for Disease Control and Prevention (2104-173).

## RESULTS

### Screening and Enrollment

**FIGURE 1.**
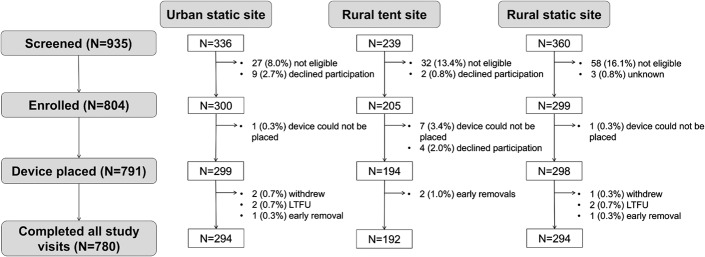
Screening and enrollment in the Malawi PrePex pilot study.

Screening outcomes are documented in Figure 1. Among 935 men screened, 804 were enrolled and 131 (14.0%) were excluded. Reasons for exclusion included HIV-positive status (n = 15), general medical exclusions (n = 5), anatomical variations or anomalies of the genitalia (n = 75), current genitourinary disease or trauma (n = 10), evidence or partial circumcision or scarification (n = 1), others (n = 7), multiple reasons (n = 4), or declined to participate (n = 11). Among 804 enrolled men, 13 (1.6%) withdrew before device placement. Postenrollment exclusions or withdrawals included cases where device sizes were too small or too large (n = 7), the client left or decided not to participate before placement (n = 4), and difficult placements due to anatomy (n = 2). Overall, 791 (84.6%) men received the PrePex device, among whom 780 (98.6%) completed all study visits. Four patients were withdrawn after early device removal or displacement, 4 participants were lost to follow-up, and 3 withdrew after scheduled removal.

### Demographics and Sexual Behavior

Most participants were young, with 86.1% under the age of 30, and represented a diverse mix of ethnic groups (Table 1). Most participants had education higher than primary school, 18.3% attended junior secondary, 37.7% attended senior secondary, and 15.9% had higher than secondary education. Men were relatively equally distributed between types of employment involving frequent lifting and moving (31.9%), mostly desk-based work (27.6%), and unemployment (34.1%). Most participants (95.3%) self-identified as Christian. Approximately one-quarter (24.3%) were married, 34.1% were in a relationship, and 41.2% reported no current sexual partner. Eighty percent of clients (n = 636) reported being sexually active. Among the 338 men who reported recent sexual activity in the last 3 months, 190 (56.2%) reported using a condom.

**TABLE 1.**
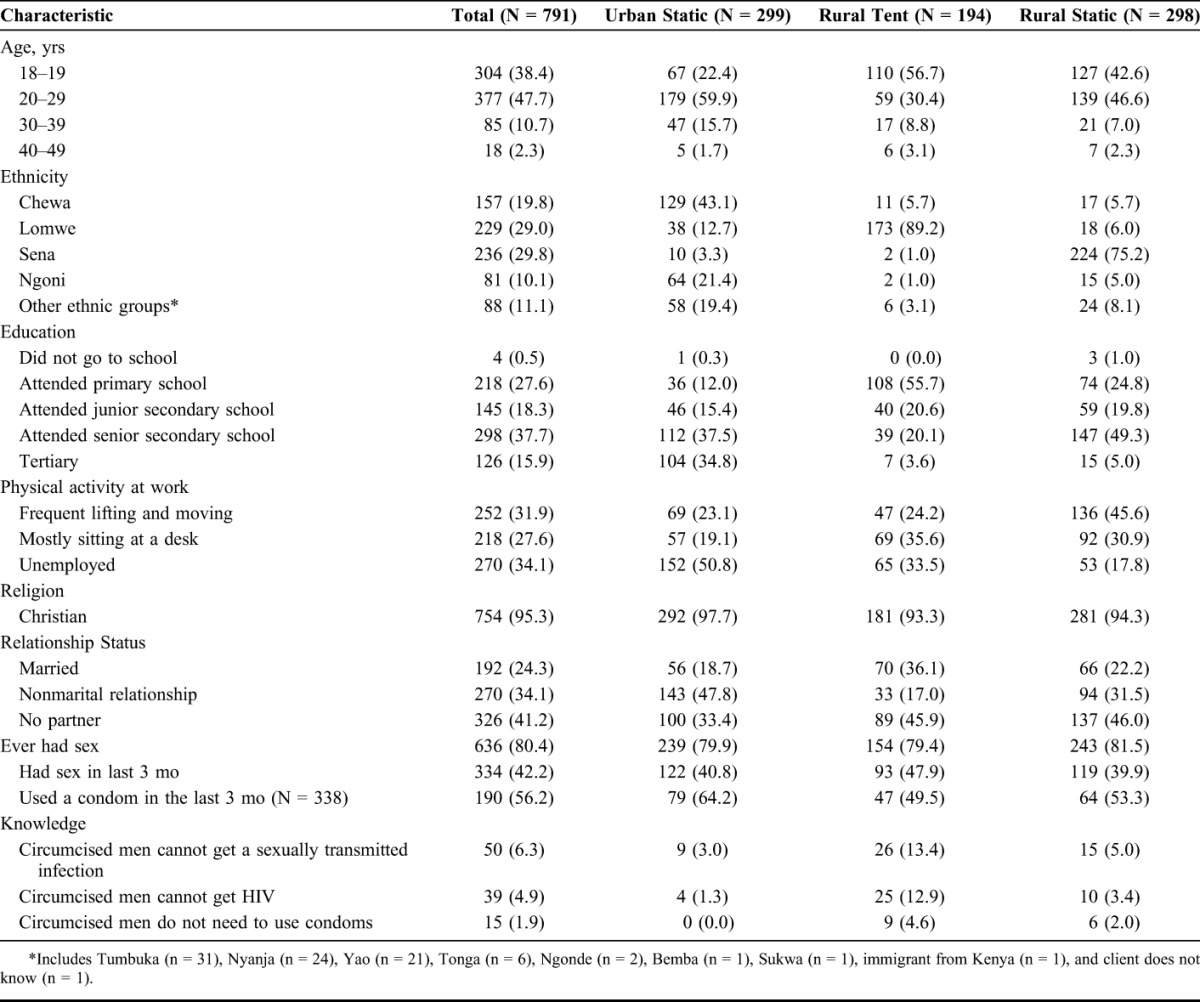
Characteristics of Men Seeking PrePex VMMC Services in Malawi Pilot Study, 2014

### Safety Outcomes

Safety outcomes are presented in Table 2. There were 56 moderate and severe AEs defined as possibly related and definitely related to the PrePex procedure, 24 (48.9%) of which were reported pain. The prevalence of AEs was 7.1% including pain (95% CI: 3.4 to 14.7) and 4.0% excluding pain (95% CI: 2.6 to 6.4). There were 53 definitely related events: 6.7% including pain (95% CI: 3.2 to 13.9) and 3.7% excluding pain (95% CI: 2.4 to 5.6).

**TABLE 2.**
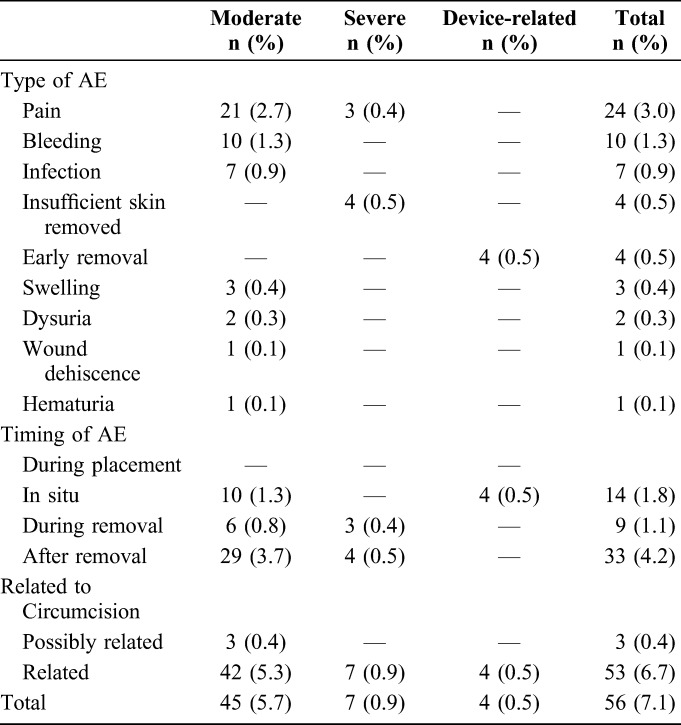
Safety—Frequency, Type, Severity, and Timing of AEs (N = 791)

Severe AEs included pain (n = 3) and insufficient skin removal requiring surgical completion (n = 4). There were 4 device-related AEs: 2 early removals occurred on day 0, 1 device displacement occurred on day 1, and 1 device displacement occurred on day 3. Among the 2 day 0 cases, 1 participant self-removed the device 8.5 hours after placement, declined surgery, and would not return despite home visits. On examination at home, the client had no physical problems. The other did not return to our site but presented at a nearby clinic without complications. The day 1 displacement presented immediately and received surgical completion. The day 3 displacement presented more than 8 hours after removal and had surgery after antibiotic treatment and observation over 48 hours at the direction of the study surgeon.

Three possible events included 2 cases of painful urination diagnosed by providers as urinary tract infections and 1 case of hematuria diagnosed as schistosomiasis. No cases of tetanus, torsion, or excessive skin removal were found. No occurrences of erectile dysfunction, scarring, or psychosocial problems were identified. Most participants (n = 731, 93.7%) had documented complete healing, defined as complete epithelialization, at day 42.

### Provider Acceptability

Providers reported relative ease of device placement and removal. Providers working in the tent site reported that it was easier to place PrePex than do surgery in the small treatment area inside the tent. Among all removals, 643 (81.7%) were rated by providers as 1 (the easiest on a scale of 1–5), 46 (5.9%) reported difficulty as a 2 or 3 of 5, and 4 (0.5%) were reported as the highest levels of difficulty as a 4 or 5 of 5. All providers reported PrePex was superior to surgical circumcision; 7 providers (58.3%) reported PrePex was “far superior,” and 5 (41.7%) reported PrePex was somewhat superior.

### Participant Acceptability: Pain, Odor, Satisfaction, and Resumption of Activities

**FIGURE 2.**
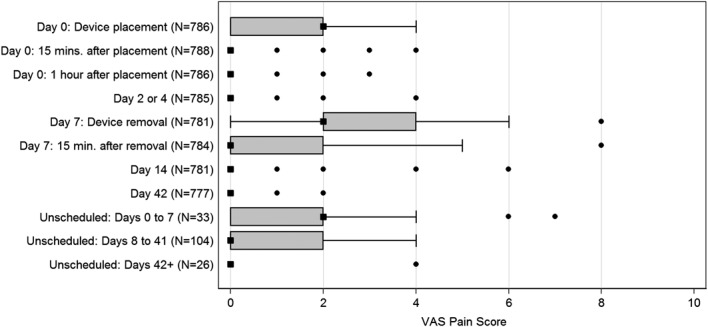
Median pain scores at study visits. Pain scores were reported using a visual analogue scale, where 0 was no pain and 10 was the highest. Scores are presented as box plots, where the boxes represent the IQR, square markers represent the median, whiskers represent values outside the IQR ± 1.5*IQR from the median, and circle markers represent values outside the whiskers. Indicators where the high and low values of the IQR are equal to the median value do not have a box.

Median levels of reported pain by participants were 2 (IQR, 2–4) during application and removal and 0 (IQR, 0–2) at all other time points (Fig. 2). Reported pain was highest during device removal and decreased 15 minutes after removal. More than half of men (51.9%) reported odor while the device was in place; however, only 17 (2.2%) stated they would not recommend the device to others because of odor. Odor was most commonly reported at day 4 (33.1%) and day 7 (48.7%) visits, compared to 4.9% of day 2 visits.

At each of the 5 follow-up visits, >90% of participants stated that they were satisfied with the procedure and results, and >97% reported that they would recommend the device to others (Table 3). Most reported that abstaining from sex was easy or very easy, with 1% (n = 8) reporting sexual activity or masturbation while the device was in place and 26 (3.3%) reporting sexual activity or masturbation during the recommended 6 weeks of abstinence. One of the displacement cases occurred after having sex with the device in place; however, the client was initially reluctant to admit this. Most clients (n = 580, 73.7%) reported that they took no time off from work or school after the procedure. Twenty participants (2.5%) took between 1 and 7 (median 3) days off, and 3 (0.4%) took more than a week. Seventy participants (8.9%) reported intentionally timing their procedure with school or work holidays.

**TABLE 3.**
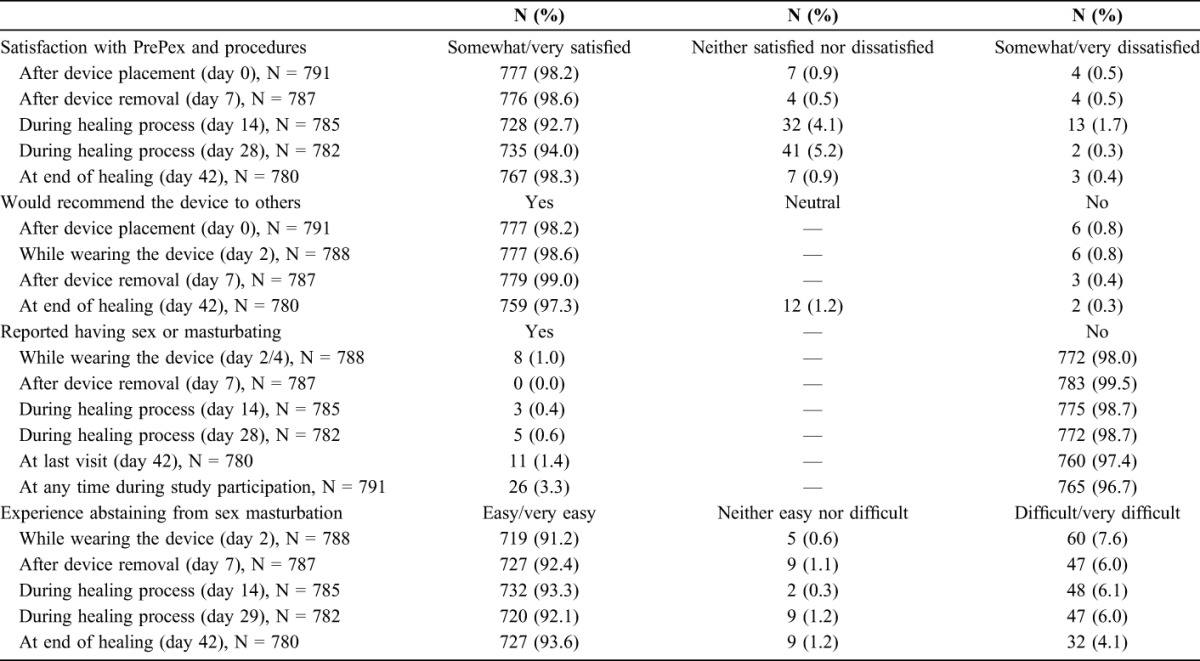
Client Acceptability and Satisfaction With the PrePex Device in Malawi

## DISCUSSION

This study found a 7.1% prevalence of AEs including pain associated with the PrePex device for adult male circumcision in Malawi. Pilot implementation studies from Uganda and Kenya have reported AEs between 1.9% and 5.9%.^15–17^ Although our AE rate was higher than the expected rates of 2.0% seen in the Rwandan trials, some of the difference can be explained by the recent inclusion of pain in the AE definitions. Studies from Rwanda^12^ and Uganda^15,16^ did not systematically include pain in their definition of a moderate or severe AE, whereas our study and the study in Kenya^17^ did. In 1 study that measured pain but did not include it in the AE definition, 15.8% reported experiencing pain of 8 or above on the visual analogue scale.^15^

Excluding pain and including possibly related events, we found 4.0% AEs. Other explanations for the higher rates may include the heightened follow-up, with only 8 clients (1.0%) lost from the study. Our program also involved secondary review of case report forms and weekly reviews of all possible events, which may have identified more cases than in some program settings. Finally, there is some variability in the literature about what constitutes an AE versus an expected side effect. The Rwanda field study listed cases of oozing, localized edema, and clear exudate as expected side effects, with 14.6% of clients experiencing these events.^12^ In our study, edema was classified as an AE (we had 3 moderate cases), and oozing or exudate may have been attributed (correctly or incorrectly) to infection.

Our findings of 4 (0.5%) device displacements and 7 severe events (0.9%) were less than or similar to those of other PrePex pilot field studies. Displacements in Uganda ranged between 0.7% and 2.0%^15,16^ and were 1.2% in Kenya.^17^ Our second most common severe event was 4 cases (0.5%) of insufficient skin removal, compared to no cases in the Uganda studies and 9 cases (2.1%) in Kenya. During a training visit, the master trainer suggested that the insufficient skin removals seemed to be associated with provider placement skills. There were no further insufficient skin removal events after a refresher training on proper placement.

The most common moderate AEs, other than pain, included infection (n = 7, 0.9%) and postremoval bleeding (n = 10, 1.3%). Providers noted some of these clients had difficulties with hygiene or applied traditional poultices, which may have contributed to topical infections. These cases also could have been misclassification of normal sloughing, which can have an appearance similar to pus. Eight of the 10 bleeding cases were clustered over 4 weeks at a single site. Although we reviewed each case, we could not identify a cause for this cluster of bleeding events. This may have been related to provider skill in cutting the elastic band at removal. In several cases, clients reported picking away scabs or exudate, which led to bleeding events.

The primary challenges were in management of displacements or early removals. Among early removal cases, 1 participant returned and had immediate surgical circumcision, 1 presented more than 8 hours after removal and had surgery after antibiotic treatment and observation, 1 declined surgery even after home visitation, and 1 did not return to our site at all. Therefore, only one of the 4 displacement clients was able to successfully access care within the recommended window of 6 hours after presentation to clinic. Access to surgical care within this window is challenging for rural settings. In 2008, Malawi had only 0.02 physicians per 1000 individuals, and these are largely concentrated in city centers. As a result, Malawi would likely not be able to comply with new general thinking that the complicated anatomy post-displacement requires advanced surgical skills. If surgical response could include any trained surgical VMMC provider, the scale-up of PrePex in Malawi would be more feasible. Furthermore, although we were able to verify that they had no further complications, the 2 early removal cases where participants refused surgery or did not return are concerning, considering the extensive patient education provided as part of this study.

There is some debate about whether immediate surgery after early removal is appropriate in all cases. In 1 case, the surgeon opted to treat with oral antibiotics and observe the participant for 48 hours after presentation as there was concern in performing surgery with the level of edema present. After 48 hours, the participant had reduced swelling and the team was able to perform the circumcision without difficulty. There may be need for additional research to determine whether delaying surgery may be appropriate in some cases and may allow nonsurgeons to safely perform the procedure after swelling has subsided.

Although our study identified no cases of tetanus, there is also concern over recent reports of 8 cases associated with VMMC in other countries.^18^ If programs must provide or confirm tetanus vaccination, this additional regimen of clinic visits may further complicate implementation. Additionally, use of traditional poultices pose risk for contamination with *Clostridium tenani*; therefore, it will be important for clinicians to strongly counsel about proper hygiene and the healing process.

Although there has been concern about odor, less than 3% of clients reported that this issue would prevent them from recommending the procedures to others. Clients reported little difficulty with abstinence; however, we found that clients were reluctant to admit to sexual activity. Therefore, the proportion of clients reporting abstinence may be an overestimate. Counseling on abstaining while the device is in place and safe resumption of sexual activity will be imperative for all programs.

Strengths of this study include little loss to follow-up and careful review of all possible events. Limitations include the withdrawal of clients from final satisfaction surveys, AEs requiring surgical circumcision and limitations in generalizability to the general male population of Malawi. Compared to the 2010 Demographic and Health Survey in Malawi, our participants had more education.^19^

In conclusion, the PrePex device for male circumcision can be safely implemented in a variety of settings in Malawi. However, there are challenges with this approach. The occurrence of rare but serious events is worrisome in the context of limited access to surgical facilities and surgical staff, the very settings where the device would also have the most immediate benefit. Training will need to be reinforced through routine supervision; and clients must be counseled appropriately in hygiene, abstinence, postplacement care, and timely presentation to clinic in the event of a displacement.
